# Nearly complete late Eocene skull from the North Pacific elucidates the cranial morphology and affinities of the penguin-like Plotopteridae

**DOI:** 10.1007/s00114-025-01977-1

**Published:** 2025-03-20

**Authors:** Gerald Mayr, James L. Goedert, Adrian Richter

**Affiliations:** 1 Ornithological Section, Senckenberg Research Institute and Natural History Museum Frankfurt/M, Senckenberganlage 25, 60325 Frankfurt am Main, Germany; 2 Burke Museum of Natural History and Culture, University of Washington, Box 353010, Seattle, WA 98195 USA; 3 Department of Messel Research and Mammalogy, Senckenberg Research Institute and Natural History Museum Frankfurt/M, Senckenberganlage 25, 60325 Frankfurt am Main, Germany

**Keywords:** Aves,, Evolution,, Fossil birds,, Morphology,, Phylogeny

## Abstract

The extinct Plotopteridae were penguin-like, wing-propelled diving birds of the North Pacific. Recently, the oldest and most complete plotopterid skull has been discovered in the late Eocene lower part of the Lincoln Creek Formation, southern Olympic Peninsula (Washington State, USA), and informs the poorly known cranial morphology of these birds. This skull is somewhat larger than previously described partial skulls from the Oligocene Pysht Formation of the northern Olympic Peninsula, from which it also differs in the shape of the nostrils. It may represent the genus *Klallamornis*, but a definitive taxonomic assignment is not yet possible. The specimen corroborates a sister group relationship of plotopterids to the suliform Suloidea and exhibits a notable character mosaic. Whereas the long rostrum most closely resembles that of the Fregatidae and some Phalacrocoracidae, the neurocranium is more similar to that of the Sulidae. An arcuate rostral ridge of the basicranium is otherwise only known from the Sphenisciformes, and a pair of prominent longitudinal ridges along the ventral surface of the rostrum is an autapomorphy of plotopterids. The small nostrils are situated at the caudal ends of conspicuous sulci, which are interpreted as vestiges of long, slit-like nostrils and are much less pronounced in extant Suliformes. Long, slit-like nostrils occur in stem group Sphenisciformes and may also have been present in stem group Fregatidae, in which case the nostrils were reduced twice within Suliformes, presumably to prevent salt water influx into the nasal cavity.

## Introduction

During the mid-Cenozoic, the North Pacific coasts around Japan and along western North America were home to an extinct group of strikingly penguin-like, wing-propelled diving birds, the Plotopteridae (Howard 1969; Olson and Hasegawa 1979, 1996; Mayr 2017, 2022). Although Howard (1969), on the basis of a fragmentary coracoid, recognized that the fossil represented a new family of wing-propelled swimming birds with similarities to darters (Anhingidae) and cormorants (Phalacrocoracidae), its precise relationships remained unknown. Since then, additional fossils have shown that these birds exhibit derived characteristics of the Suliformes, the clade including the Fregatidae (frigate birds) and Suloidea (Sulidae [gannets and boobies], Anhingidae, and Phalacrocoracidae). However, the affinities of plotopterids within this clade are debated, and whereas some authors deemed them most closely related to the Phalacrocoracidae and Anhingidae (Olson and Hasegawa 1979, 1996; Smith 2010), others considered them stem group representatives of the Suloidea (Mayr et al. 2015, 2021). It was also proposed that plotopterids are the sister taxon of the Sphenisciformes (penguins), and that the latter evolved from a suliform ancestor (Mayr 2005). This hypothesis is not supported by analyses of nuclear sequence data, which consistently support a sister group relationship between the Sphenisciformes and Procellariiformes and find the former to be only distantly related to the Suliformes (e.g., Prum et al. 2015; Kuhl et al. 2021; Stiller et al. 2024).

Most North American plotopterids stem from late Eocene and Oligocene strata in Washington State (USA) and were assigned to the taxa *Tonsala*, *Phocavis*, *Olympidytes*, and *Klallamornis* (Olson 1980; Goedert 1988; Mayr et al. 2015; Mayr and Goedert 2016, 2018, 2022), whereas plotopterids from the late Eocene and Oligocene of Japan are classified into *Copepteryx*, *Hokkaidornis*, *Stenornis*, and *Empeirodytes* (Olson and Hasegawa 1996; Sakurai et al. 2008; Ohashi and Hasegawa 2020); furthermore, Mori and Miyata (2021) assigned a fragmentary distal tibiotarsus to *Olympidytes*. Some of these taxa are represented by partial skeletons and multiple isolated bones, and the postcranial anatomy of plotopterids is comparatively well-known. However, published data on their skull morphology is restricted to two fragmentary Oligocene specimens from the Pysht Formation on the north side of the Olympic Peninsula, and neither preserve complete beaks nor show many details of the neurocranium (Mayr et al. 2015). Undescribed partial plotopterid skulls from Japan were figured by earlier authors, but have not yet been described (Hasegawa et al. 1979; Kawabe et al. 2014; Knoll and Kawabe 2020); virtual endocasts showed the brain morphology of these Japanese plotopterids to be similar to that of crown group Sphenisciformes (Kawabe et al. 2014).

Here, we report a well-preserved plotopterid skull from late Eocene strata of the Lincoln Creek Formation on the southern side of the Olympic Peninsula in Washington State, USA. It is the oldest known plotopterid skull and one of the most complete and best preserved skulls of a Paleogene seabird in general. The new specimen exhibits a notable character mosaic, which informs the phylogenetic affinities and ecology of plotopterids, and it is associated with a partial humerus allowing some comparisons with previously described plotopterids that are based on postcranial bones.

## Material and methods

The fossils and the extant comparative material are in the collections of the Canterbury Museum, Christchurch, New Zealand (CM), the Natural History Museum of Los Angeles County, Los Angeles, CA, USA (LACM), the Senckenberg Research Institute Frankfurt, Germany (SMF), and the University of Canterbury, Christchurch, New Zealand (UC). Comparisons were made with skulls of the following extant species (nomenclature follows the IOC World Bird List at https://www.worldbirdnames.org): Fregatidae: *Fregata magnificens*; Sulidae: *Morus bassanus*, *M. capensis*, *M. serrator*, *Sula dactylatra*, *S. leucogaster*, *S. nebouxii*, *S. sula*, *S. variegata*; Anhingidae: *Anhinga anhinga*, *A. rufa*; Phalacrocoracidae: *Microcarbo africanus*, *M. melanoleucos*, *M. pygmaeus*, *Nannopterum auritum*, *N. brasilianum*, *N. harrisi*, *Gulosus aristotelis*, *Poikilocarbo gaimardi*, *Leucocarbo atriceps*, *L. bougainvilliorum*, *L. magellanicus*, *L. melanogenis*, *Urile pelagicus*, *U. penicillatus*, *Phalacrocorax capensis*, *P. carbo*, *P. nigrogularis*, *P. punctatus*, *P. varius.*

The fossil was collected with hand tools (geology picks). It was prepared with a 4% solution of formic acid buffered with tricalcium phosphate. For better handling of the specimen, a screw thread was added (embedded in resin matrix reinforced with glass beads), which can be attached to a stand. Stratigraphy is based on Rau (1966), and stratigraphic position was determined with a tape measure, with the measurement then corrected for dip.

Micro-CT scans were performed in a customized version of the Tomo Scope XS Plus 200 (Werth Messtechnik, Gießen, Germany) with 160 kV voltage, 30 W power, an exposure time of 1000 ms, and 1600 projections. Three images were averaged to reduce noise. To cover the whole specimen, five individual scans were taken and stitched automatically during the reconstruction process in the manufacturer’s WinWerth software. The volume with 38.35 µm voxel size was initially exported as a 32-bit float raw image.rek file which was transformed to 16-bit with the WinWerth Software, before cropping and transformation to 8-bit data in Avizo 3D (Thermo Fisher Scientific, USA), from where it was exported as.tif image stack. The image stack was imported to VG Studio Max (Volume Graphics, Germany) for volume rendering. Unfortunately, low differences in radiodensity between the fossilized bones and the surrounding matrix led to limited contrast. This prevented digital removal of the matrix, so that the rendered images mainly show surface structures.

Based on the new skull and the data on Paleocene penguins in Mayr et al. (subm.), we emended the matrix of Mayr et al. (2015). Six scorings were added or corrected for the Plotopteridae (character numbers refer to the matrix in Mayr et al. 2015): 1:1, 3:1 (corrected), 5:1, 6:1, 19:1, 20:1; nine scorings were added for *Muriwaimanu*: 1:0, 3:2, 5:1, 6:0, 13:0, 14:0, 17:0, 32:0, 81:0.

Seven characters were newly added to the matrix of Mayr et al. (2015): (95) Praemaxilla, ventral surface of tip densely pitted with sensory foramina: no (0), yes (1); this character was scored present in the Plotopteridae, Sulidae, Phalacrocoracidae, Anhingidae, Sphenisciformes, Diomedeidae, and Threskiornithidae; it was scored as unknown in *Limnofregata*, *Muriwaimanu*, and *Icadyptes*, and as absent in all other taxa. (96) Fossa temporalis medially extensive and rostrocaudally elongated: no (0), yes (1); this character was scored present in the Plotopteridae and Phalacrocoracidae, and it was scored as absent in all other taxa. (97) Caudal portion of brain cavity markedly laterally bulging: no (0), yes (1); this character was scored present in the Anhingidae and Phalacrocoracidae, and it was scored as absent in all other taxa. (98) Tubercula basilaria strongly developed and ventrally prominent: no (0), yes (1); this character was scored present in the Plotopteridae, Sulidae, Anhingidae, Phalacrocoracidae, and Sphenisciformes, and it was scored as unknown in *Limnofregata* and as absent in all other taxa. (99) Skull, basicranial area with conspicuous, arcuate rostral ridge, which delimits an essentially flat lamina parasphenoidalis: absent (0), present (1); this character was scored present in the Plotopteridae and Sphenisciformes, and as absent in all other taxa. (100) Humerus, crista bicipitalis proximodistally long (longer than the dorsoventral length of the caput humeri): no (0), yes (1); this character was scored present in the Pelecanidae, Sulidae, Anhingidae, and Phalacrocoracidae and absent in all other taxa. (101) Pelvis, spina dorsolateralis pelvis strongly elongated and narrow (more than two times longer than it is dorsoventrally wide): no (0), yes (1); this character was scored present in the Plotopteridae, Sulidae, Anhingidae, and Phalacrocoracidae; it was scored as unknown in *Icadyptes* and as absent in all other taxa.

The analysis was run with the heuristic search mode of NONA 2.0 (Goloboff 1993) through the WINCLADA 1.00.08 interface (Nixon 2002), using the commands hold 10000, mult*1000, hold/10, and max*. Bootstrap support values were calculated with 1000 replicates, ten searches holding ten trees per replicate, and TBR branch swapping without max*.

## Systematic paleontology

Aves Linnaeus, 1758

Suliformes Sharpe, 1891

Plotopteridae Howard 1969

Tonsalinae Mayr and Goedert 2018

Gen. et sp. indet.

### Referred specimen

**Fig. 1:**
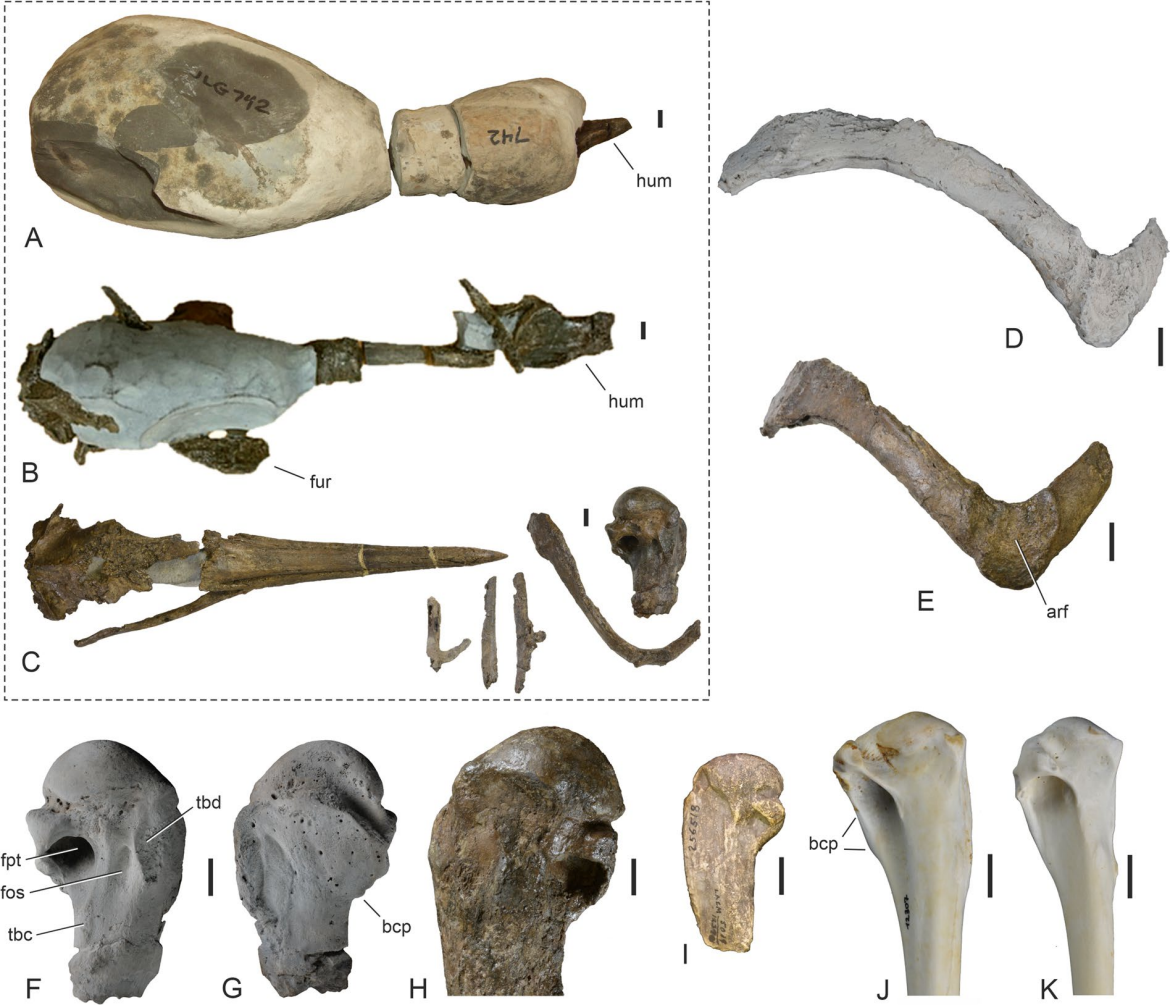
Different preparation stages of the new plotopterid specimen (SMF Av 671) from the late Eocene of the Lincoln Creek Formation (**A**, **B**, **C**), as well as details of the associated partial furcula (**D**, **E**), and the proximal end of the humerus in comparison to that of other plotopterids from the Olympic Peninsula and extant Suliformes (**F**, **G**, **H**, **I**, **J**, **K**). **A** The three nodules, which contained the specimen before preparation (the bone exposed at the tip of one of the nodules is the humerus shaft and not the beak; note that the specimen is shown in a different view than in **B** and **C**, so that the humerus shaft is seen in lateral view and appears narrower than in **B** and **C**). **B** The specimen at an intermediate stage of acid preparation, which exposed some of the bones. **C** The fully prepared specimen, which includes a skull, three rib fragments and a partial furcula, as well as a proximal humerus. **D, E** Extremitas sternalis of the furcula of SMF Av 671 in craniolateroventral (**D**) and cranioventral (**E**) view; in **D** the specimen was coated with ammonium chloride. **F**, **G** Proximal end of right humerus of SMF Av 671 in cranial (**F**) and caudal (**G**) view; coated with ammonium chloride. **H** ?*Klallamornis clarki*, proximal end of left humerus (SMF Av 664) from the Lincoln Creek Formation in caudal view. **I** *Tonsala hildegardae*, proximal end of left humerus (cast of holotype, LACM 123791) from the Pysht Formation in caudal view. **J** The extant *Morus bassanus* (Sulidae, SMF 12307), right humerus in caudal view. **K** The extant *Phalacrocorax carbo* (Phalacrocoracidae, SMF 2939), right humerus in caudal view. arf, articular facet for apex carinae of sternum; bcp, crista bicipitalis; fos, fossa between fossa pneumotricipitalis and tuberculum dorsale; fpt, fossa pneumotricipitalis; fur, furcula; hum, humerus; tbc, tubercle for insertion of musculus latissimus dorsi, pars cranialis; tbd, tuberculum dorsale. The scale bars equal 10 mm

SMF Av 671 (Fig. 1C; skull, some rib fragments, partial furcula, proximal end of right humerus); from three associated calcareous concretions found in situ by JLG in July 2023.

### Locality and horizon

East bank of the middle fork of the Satsop River, approximately 53.2 m above the contact with the Crescent Formation basalt, 47.30201° N, 123.46508° W, Mason County, Washington State, USA; lower part of the Lincoln Creek Formation, late Eocene (Rau 1966; Prothero and Armentrout 1985).

### Measurements (in mm)

Skull, maximum total length, 277.7; rostrum, length, 176.4. Humerus, maximum dorsoventral width of proximal end, 46.5.

### Taxonomic remarks

**Fig. 2:**
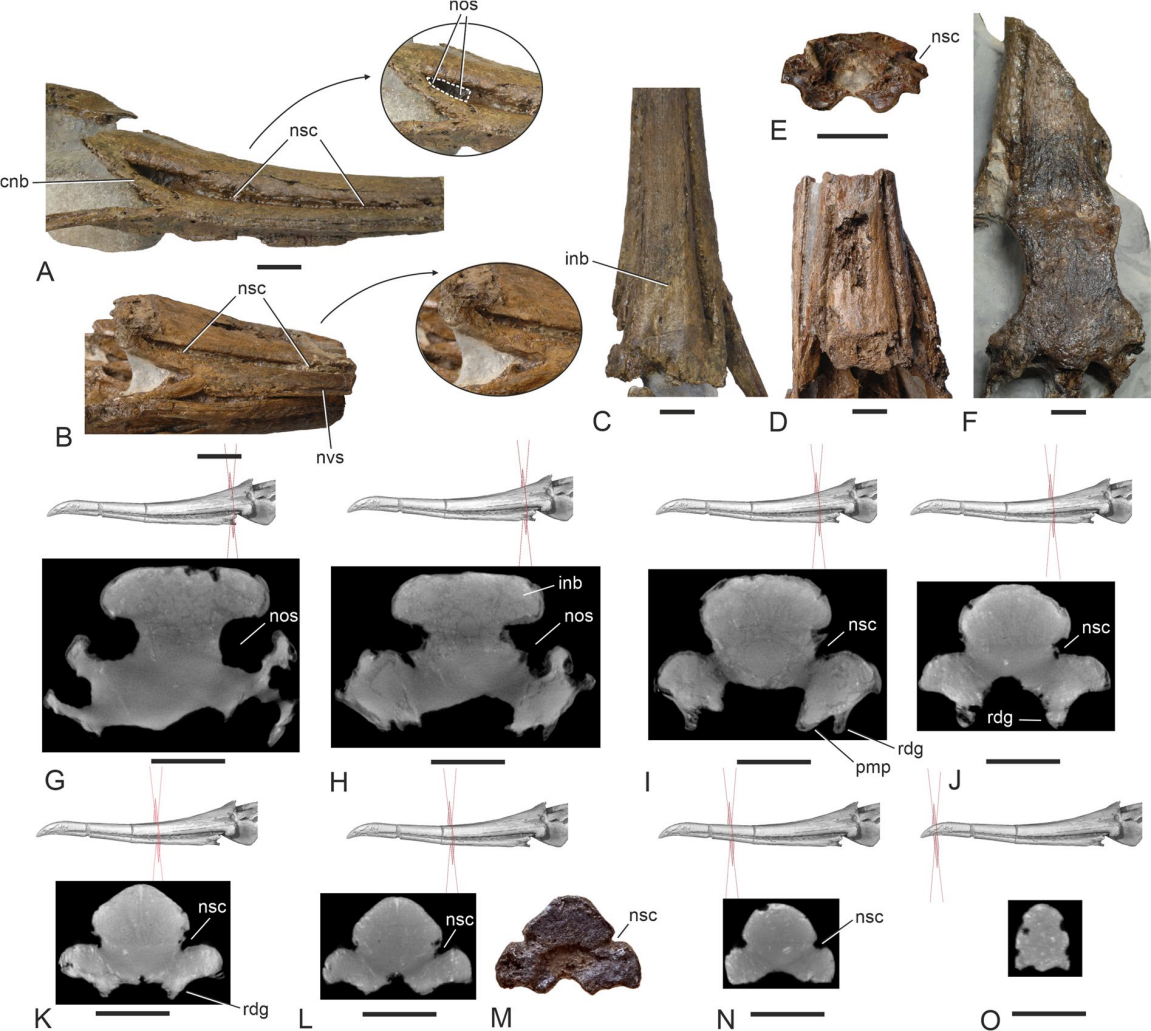
Details of the nasal region and beak of plotopterids from the Olympic Peninsula. **A** Detail of the nasal region of the new plotopterid skull from the late Eocene of the Lincoln Creek Formation (SMF Av 671) in right lateral view; the dotted line denotes the outline of the nostril. **B** Detail of the nasal region of the plotopterid specimen SMF Av 599 from the Oligocene of the Pysht Formation, which was described by Mayr et al. (2015) in right lateral view. **C**, **D**, **E**, **F** Detail of the nasal region of plotopterids from the Olympic Peninsula in dorsal view (**C**, SMF Av 671; **D**, **E**, SMF Av 599 [**E** shows the cross section of the tip of the rostrum at the breakage, which is in approximately the position of the cross section shown in **K**]; **F** SMF Av 600 from the Pysht Formation [see Mayr et al. 2015]). **G**‒**O** Cross-sections of the rostrum of SMF Av 671 at different levels; **G**, **H**, **I**, **J**, **K**, **L**, **N**, **O** are CT scans, **M** shows the rostrum at the breakage just rostral to the cross section depicted in **L**. The arrows in **A** and **B** denote enlarged details. cnb, caudal nasal bar; inb, internarial bar; nos, nostril; nsc, nasal sulcus; nvs, neurovascular sulcus; pmp, processus maxillopalatinus; rdg, ventral ridge of rostrum. The scale bars equal 10 mm

Two previously described partial plotopterid skulls from the latest early Oligocene/early late Oligocene Pysht Formation, catalogued as SMF Av 599 and SMF Av 600 (Fig. 2B, D, F; Mayr et al. 2015), are somewhat smaller than SMF Av 671 and have a narrower interorbital section of the frontals (the minimum width is 25.5 mm in SMF Av 600 versus 30.3 mm in SMF Av 671); the internarial bar has a narrower caudal end and its caudal section tapers less rostrally (the minimum width of the internarial bar at the caudal terminus of the nasal sulcus is 20.2 mm in SMF Av 600 and 20.7 mm in SMF Av 599, whereas it is 25.6 mm in SMF Av 671). More importantly, however, the two specimens from the Pysht Formation differ from the new skull in that the caudal portions of the nostrils are dorsoventrally narrower and more slit-like (Fig. 2A, B).

Based on size comparisons with extant Sulidae, at a time when fewer plotopterid fossils from the Olympic Peninsula were available for comparisons, the skulls from the Pysht Formation were referred to *Tonsala hildegardae* (Mayr et al. 2015; see also Mayr and Goedert 2022: tab. 1). However, the proximal humerus of SMF Av 671 (Fig. 1F, G) is distinctly larger than that of *T. hildegardae*, which is one of the smallest known plotopterids (Fig. 1I; the proximal humerus of *T. hildegardae* has a width of only 27.9 mm; Olson 1980). The partial skulls from the Pysht Formation are only slightly smaller than the new skull, thus they are much too large to be conspecific with *T. hildegardae*. Most likely, therefore, these skulls belong to one of the two species currently assigned to the larger plotopterid taxon *Klallamornis*, likely either *K. abyssa* or *K. buchanani* found in the same part of the Pysht Formation (Mayr and Goedert 2016, 2022). *Phocavis maritima*, which is only known from the holotype tarsometatarsus from the late Eocene section of the Keasey Formation in Oregon, is likewise too small to be conspecific with SMF Av 671.

Three plotopterid specimens were previously known from the Lincoln Creek Formation (Mayr and Goedert 2022). One of these is the holotype of *Olympidytes thieli*, which consists of well-preserved leg elements, found in the lower part of the formation in southwest Washington State (Mayr and Goedert 2016); these bones are too small to be conspecific with SMF Av 671 (in plotopterids, the proximal end of the humerus is only slightly wider than the proximal end of the femur [e.g., Mayr and Goedert 2022: Fig. 3], whereas the proximal humerus of SMF Av 671 is nearly twice as wide as the proximal femur of the *O. thieli* holotype [46.5 mm versus 28.5 mm]). Another plotopterid fossil from the lower part of the Lincoln Creek Formation, found as float on the Middle Fork of the Satsop River (therefore, its exact stratigraphic position is unknown), consists of the proximal ends of two very large humeri and an associated vertebra; this specimen was assigned to ?*K. clarki*, which is the largest known plotopterid from the Olympic Peninsula (Mayr and Goedert 2022). A further isolated thoracic vertebra was also referred to ?*K*. *clarki* by Mayr and Goedert (2022, and references therein) and was found very close to where the new skull stems from. With a proximal width of 56.0 mm, the aforementioned partial humeri assigned to ?*K. clarki* are significantly larger than that of SMF Av 671, but we cannot exclude the possibility that plotopterids showed a pronounced sexual dimorphism in size, as do extant Phalacrocoracidae. The more complete of the previously described humeri from the Lincoln Creek Formation lacks the fossa between the fossa pneumotricipitalis and the tuberculum dorsale, even though the bone surface of this specimen is very eroded (Fig. 1H). This fossa actually distinguishes SMF Av 671 from all other plotopterids for which the proximal end of the humerus is known.

**Fig. 3:**
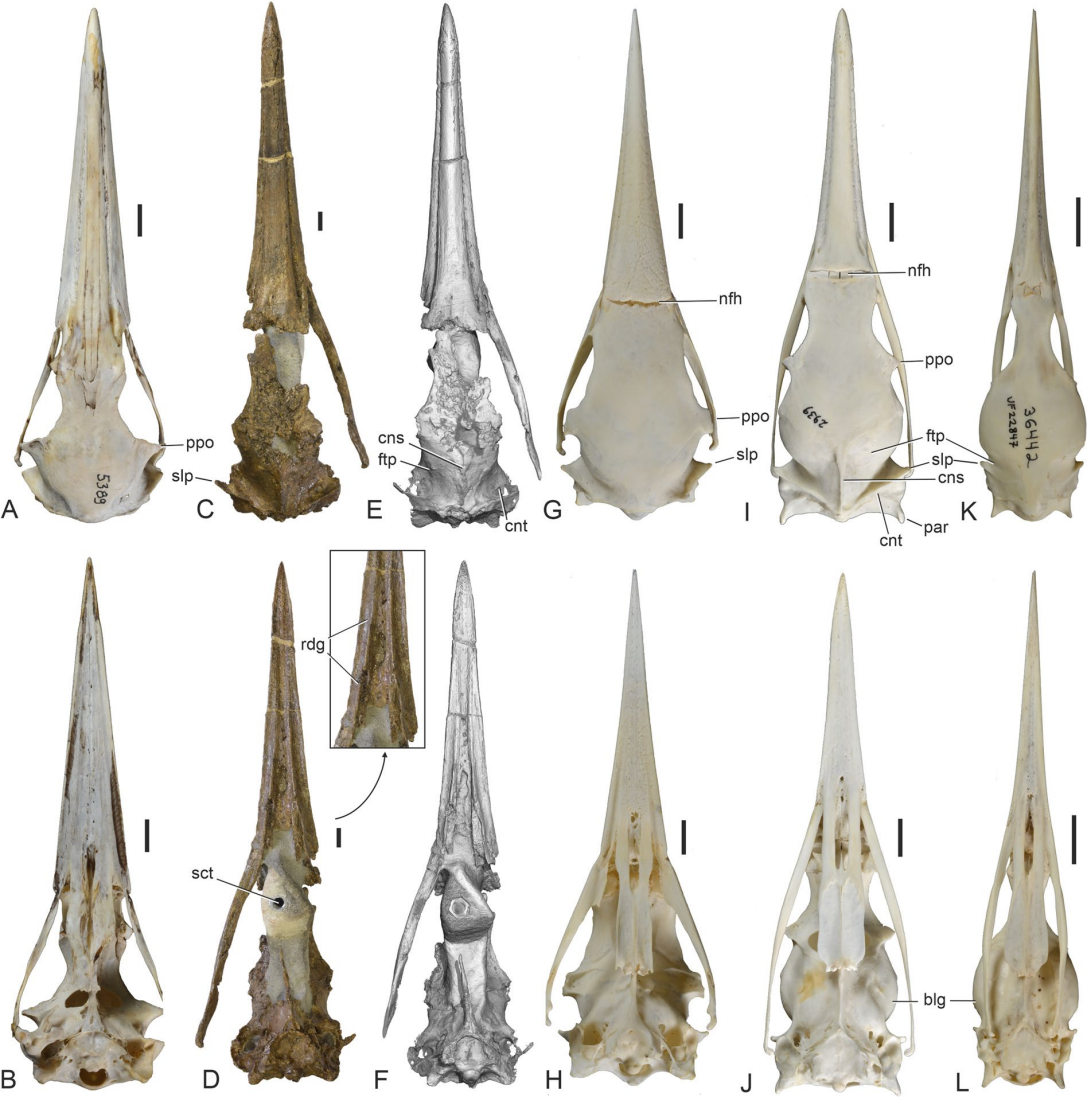
Skulls of the plotopterid from the late Eocene of the Lincoln Creek Formation and extant suliform birds in dorsal (upper row) and ventral (lower row) view. **A**, **B** The extant *Fregata magnificens* (Fregatidae, SMF 5389). **C**‒**F** The new plotopterid skull (SMF Av 671); **E** and **F** are micro-CT rendered image (the caudalmost tip of the jugal bar was outside the scanning range). **G**, **H** The extant *Sula variegata* (Sulidae, SMF 13639). **I**, **J** The extant *Phalacrocorax carbo* (Phalacrocoracidae, SMF 2939). **K**, **L** The extant *Anhinga anhinga* (Anhingidae, SMF 9967). blg, lateral bulge of neurocranium; cns, crista nuchalis sagittalis; cnt, crista nuchalis transversa; ftp, fossa temporalis; nfh, nasofrontal hinge; par, processus paroccipitales, ppo, processus postorbitalis; rdg, ventral ridge of rostrum; sct, screw thread for the assembly of the specimen on a stand; slp, shelf-like lateral projection formed by crista nuchalis transversa. The scale bars equal 10 mm

The disparate shapes of the nostrils (Fig. 2A, B) indicate that the skulls from the Lincoln Creek and Pysht formations are from different species. The holotypes of *K. abyssa* and *K. buchanani* stem from the Pysht Formation (Mayr and Goedert 2022) and the humeri of both species are as yet unknown. *Klallamornis buchanani* is somewhat smaller than *K. abyssa*, so that SMF Av 671 may belong to the latter species and the partial skulls from the Pysht Formation to the former (not considering the great difference in geochronological age between SMF Av 671 and the specimens from the Pysht Formation). However, the size difference is not large enough for an unambiguous referral of any of the skulls to either species, especially if the possibility of sexual dimorphism in size (which is particularly pronounced in the Phalacrocoracidae) is taken into account. It is also well possible, if not likely, that there was a higher diversity of plotopterids in the northeastern Pacific than what is apparent from the currently known specimens. For example, *Phocavis maritimus* from the late Eocene or earliest Oligocene Keasey Formation in northwestern Oregon (Goedert 1988) is approximately the same age as the fossils from the lowermost part of the Lincoln Creek Formation, and it was much smaller than either *Olympidytes* or *Klallamornis*. As a consequence, at this time we are unable to assign SMF Av 671 to any particular species.

### Description and comparisons

**Fig. 4:**
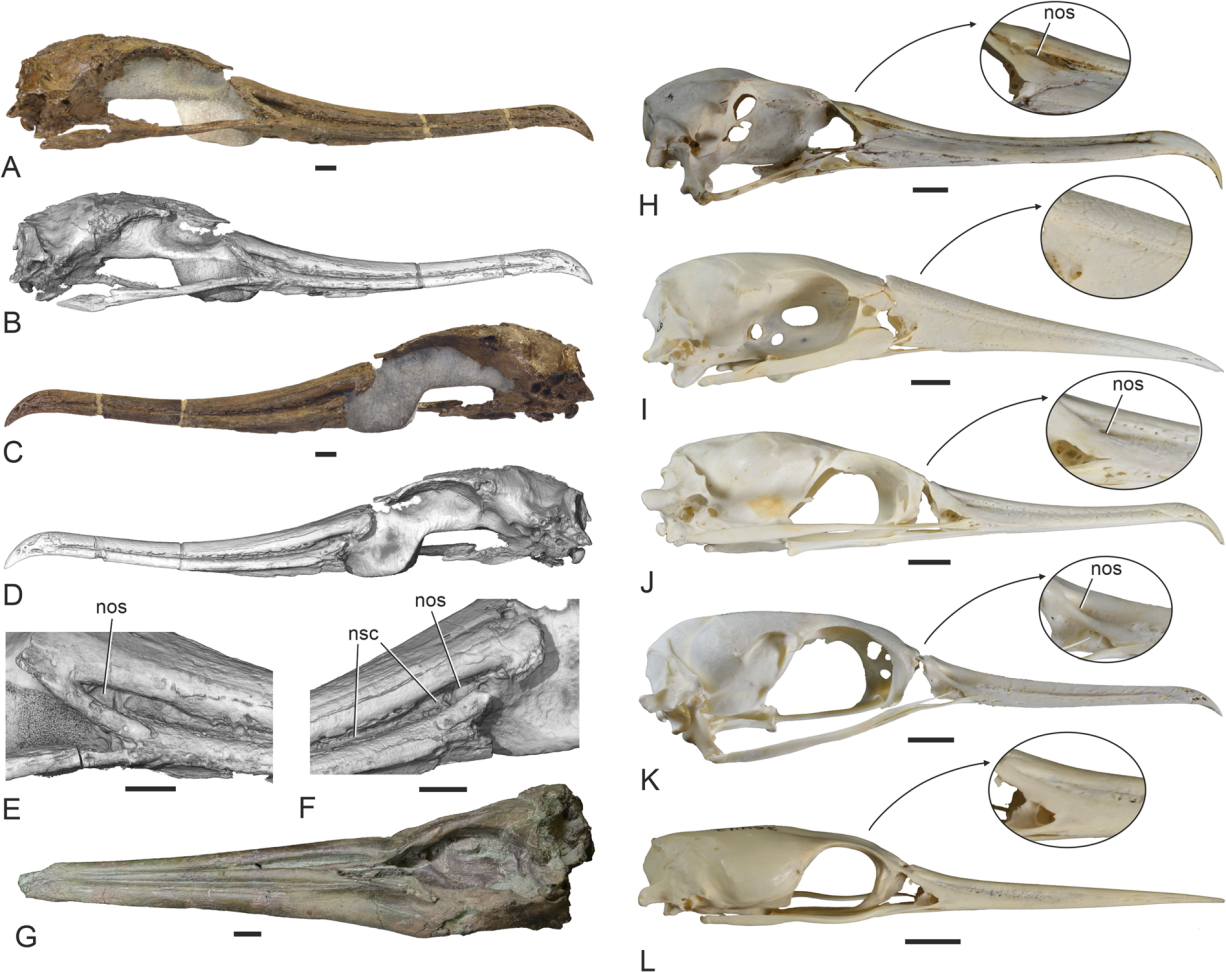
Skulls of the plotopterid from the late Eocene of the Lincoln Creek Formation, extant Suliformes, other Plotopteridae, and a stem group representative of the Sphenisciformes. **A, B**, **C**, **D** The new plotopterid skull (SMF Av 671) in right lateral (**A**, **B**) and left lateral (**C**, **D**) view; **B** and **D** are micro-CT rendered image (the caudalmost tip of the jugal bar was outside the scanning range). **E**, **F** Details of the nasal region of SMF Av 671 in right (**E**) and left (**F**) lateral view. **G** Skull of the stem group sphenisciform *Sequiwaimanu rosieae* (holotype, CM 2016.6.1) from the Paleocene of New Zealand (left lateral view). **H** The extant *Fregata magnificens* (Fregatidae, SMF 5389; right lateral view). **I** The extant *Sula variegata* (Sulidae, SMF 13639; right lateral view). **J** The extant *Phalacrocorax carbo* (Phalacrocoracidae, SMF 2939; right lateral view). **K** The extant *Leucocarbo atriceps* (Phalacrocoracidae, SMF 13265; right lateral view). **L** The extant *Anhinga anhinga* (Anhingidae, SMF 9967; right lateral view). The arrows in **H**, **I**, **J**, **K**, **L** denote enlarged details of the nasal region. nos, nostril; nsc, nasal sulcus. The scale bars equal 10 mm

The skull (Figs. 3 and 4) is largely complete except for the lack of the left jugal arch and the palatine bones, and some predepositional damage (bioerosion) to the neurocranium. It is broken at the nasofrontal hinge and the rostrum and neurocranium are slightly displaced relative to each other. This damage occurred prior to being fossilized and is likely to be due to marine predators or scavengers (see Mayr et al. 2015: 5). Considerable disturbance of the carcass is also suggested by the fact that the proximal end of the humerus was situated near the tip of the rostrum and the fragment of the furcula was preserved near the right orbit (Fig. 1B).

**Fig. 5:**
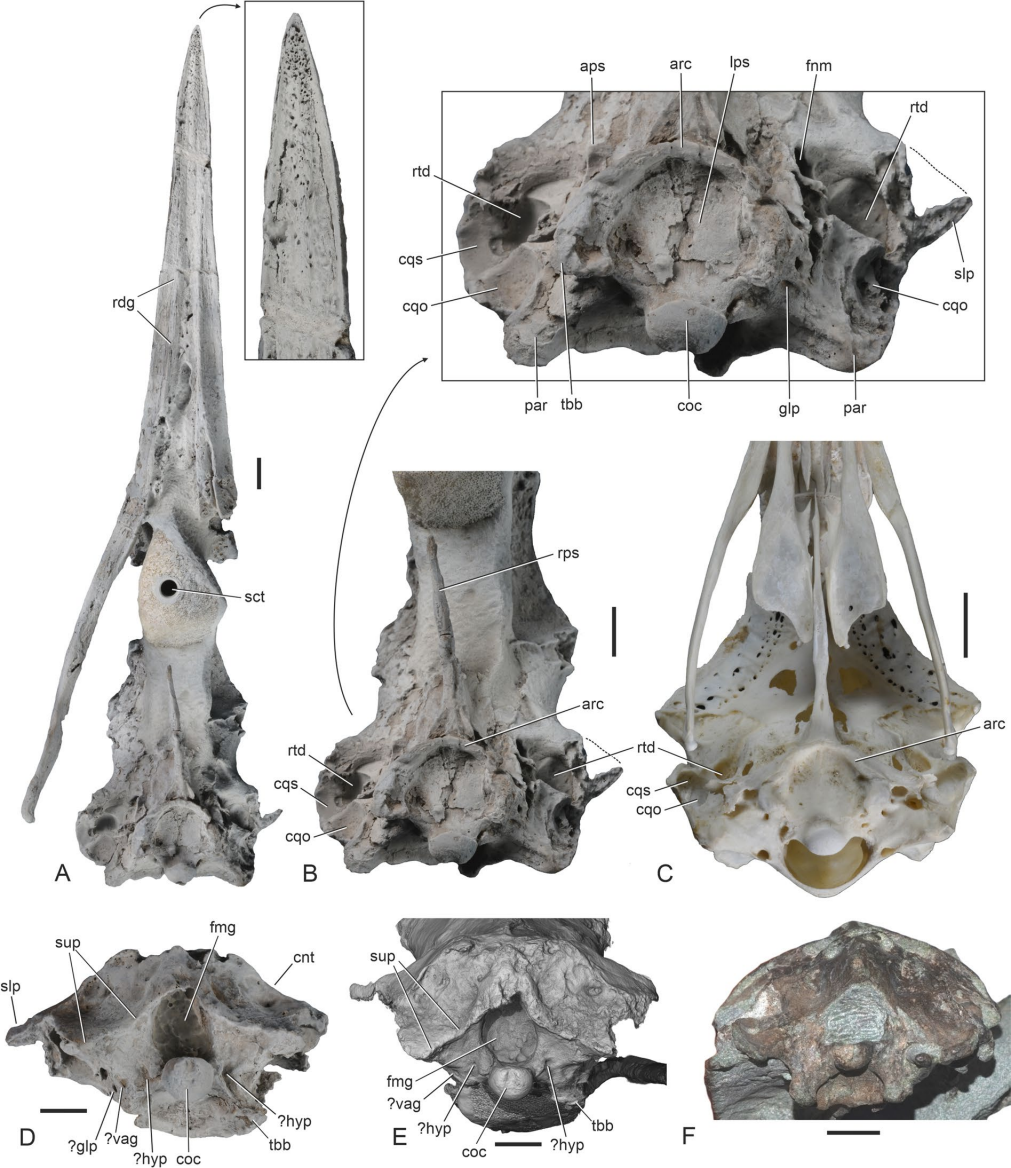
The plotopterid skull from the late Eocene of the Lincoln Creek Formation in comparison to extant and fossil Sphenisciformes. **A** The new plotopterid skull (SMF Av 671) in ventral view; coated with ammonium chloride, the arrow denotes and enlarged detail of the tip of the rostrum. **B** The neurocranium of SMF Av 671 in ventral view; coated with ammonium chloride, the arrow denotes and enlarged detail of the basicranial area, and the dotted lines indicate the reconstructed shape of the shelf-like lateral projection formed by crista nuchalis transversa. **C** Neurocranium of the extant *Megadyptes antipodes* (Spheniscidae, SMF 22581) in ventral view. **D**, **E** Neurocranium of SMF Av 671 in caudal view; **D** was coated with ammonium chloride, **E** is a micro-CT rendered image. **F** Neurocranium of the stem group Sphenisciform *Muriwaimanu tuatahi* (UC 22078) from the Paleocene of New Zealand in caudal view. aps, ala parasphenoidalis; arc, arcuate rostral ridge; cnt, crista nuchalis transversa; cqo, cotyla quadratica otica; cqs, cotyla quadratica squamosa; coc, condylus occipitalis; fmg, foramen magnum; fnm, foramen nervi maxillomandibularis; glp, foramen nervi glossopharyngealis; hyp, foramina nervi hypglossi; lps, lamina parasphenoidalis; par, processus paroccipitalis; rdg, ventral ridge of rostrum; rps, rostrum parasphenoidalis; rtd, recessus tympanicus dorsalis; sct, screw thread for the assembly of the specimen on a stand; slp, shelf-like lateral projection formed by crista nuchalis transversa; sup, ventral margin of os supraoccipitale; tbb, tuberculum basilare; vag, foramen nervi vagi. The scale bars equal 10 mm

The skull is the size of that of the Antipodean albatross (*Diomedea antipodensis*), which is one of the largest extant seabirds. The long rostrum measures about 60% of the entire skull. It was preserved within three consecutively associated—and presumably putrefaction-induced (e.g., Yoshida et al. 2015)—concretions, and two portions of the rostrum were re-assembled after acid-preparation of the fossil (Fig. 1A‒C). In its proportions and shape, the rostrum of SMF Av 671 most closely resembles that of the Fregatidae (which is, however, proportionally longer and has a more deeply hooked tip) and the Phalacrocoracidae (which is straighter and proportionally shorter in most species). The culmen (dorsal ridge) is sigmoidally curved as in the Fregatidae, but the praemaxilla is not as deeply hooked as it is in frigatebirds. The tip of the praemaxilla has a densely pitted ventral surface as in the Sphenisciformes and all Suliformes to the exception of the Fregatidae (Fig. 5A; du Toit et al. 2024). The pits in SMF Av 671 are continuous with minute grooves or sulci and thus differ from borings made by the bone-eating annelid *Osedax*, which would be straight-sided and appearing as if made by a drill (Kiel et al. 2011).

Based on fragmentary plotopterid skulls from the Pysht Formation, the nostril of plotopterids was considered long and slit-like (Mayr et al. 2015). However, the new skull shows that it is a small opening, which has a length of 11.5 mm and is situated at the caudal terminus of a marked nasal sulcus; this opening is proportionally larger than in crown group Suloidea and of similar size to the nostril of the Fregatidae (Fig. 4). Rostral to the narial opening, the nasal sulcus is medially closed by a thin osseous sheet. The nasal sulcus of SMF Av 671 is dorsoventrally deeper and less “slit-like” in its caudal portion than in previously described fragmentary plotopterid skulls from the Pysht Formation (Fig. 2A, B; Mayr et al. 2015). In extant Suloidea, this sulcus is much shallower, even though its caudal portion is well marked in some Phalacrocoracidae (*Leucocarbo* spp., *Poikilocarbo gaimardi*). As in crown group Suloidea, there are neurovascular foramina along the margins of the nasal sulcus of SMF Av 671. The culmen and the tomia of SMF Av 671 are more curved than in a partial rostrum from the Oligocene of Japan, which was likened to the Plotopteridae by Kawabe et al. (2021); furthermore, unlike in the latter specimen, the nasal sulcus of SMF Av 671 becomes dorsoventrally deeper towards the caudal end of the rostrum.

**Fig. 6:**
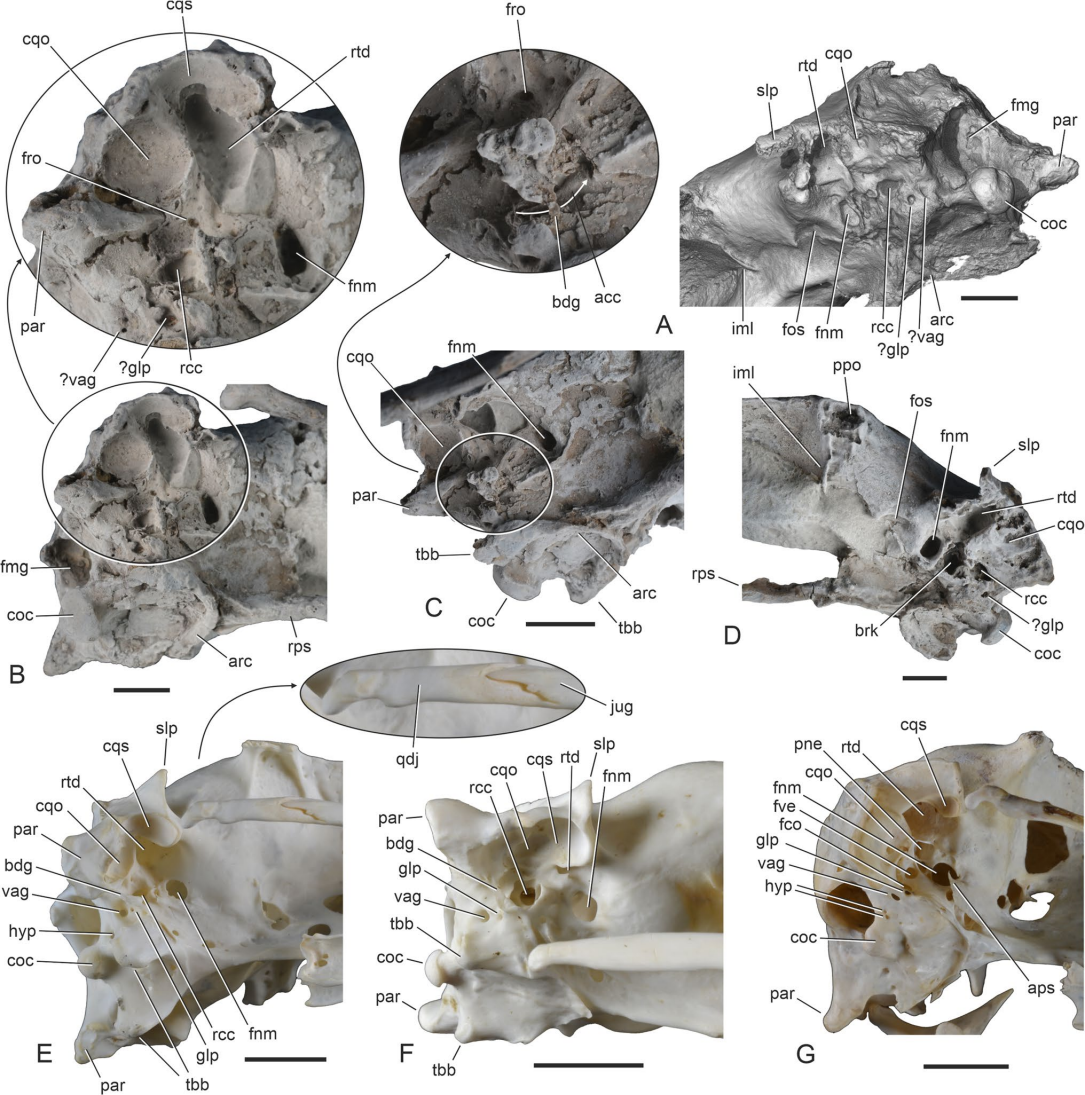
Details of the otic region of the plotopterid from the late Eocene of the Lincoln Creek Formation and extant Suliformes. **A**, **B**, **C**, **D** The new plotopterid (SMF Av 671) in left caudoventrolateral (**A**) right ventrolateral (**B**, **C**), and left ventrolateral (**D**) view; **A** is a micro-CT rendered image, in **B**, **C**, **D,** the fossil was coated with ammonium chloride; the white arrow in **C** indicates the course of the sulcus for arteria carotis cerebralis, the black arrows denote enlarged details. **E**, **F**, **G** Otic region (ventrolateral view) of **E** *Sula variegata* (Sulidae, SMF 13639; right side, note that—unlike in other neornithine birds, and as shown in the enlarged detail—os jugale and os quadratojugale are not co-ossified and separated by a suture), **F** *Phalacrocorax carbo* (Phalacrocoracidae, SMF 2939; right side), and **G** *Fregata magnificens* (Fregatidae, SMF 5389; left side mirrored). acc, sulcus for arteria carotis cerebralis; aps, ala parasphenoidalis; arc, arcuate rostral ridge; bdg, bridge above sulcus for arteria carotis cerebralis; brk, breakage; coc, condylus occipitalis; cqo, cotyla quadratica otica; cqs, cotyla quadratica squamosa; fco, fenestra cochleae; fmg, foramen magnum; fnm, foramen nervi maxillomandibularis; fos, fossa indicating a muscle attachment site; fro, foramen for ramus occipitalis of arteria ophthalmica externa; fve, fenestra vestibuli; glp, foramen nervi glossopharyngealis; hyp, foramen nervi hypoglossi; iml, intermuscular line (rostral delimitation of musculus pseudotemporalis superficialis); jug, os jugale; par, processus paroccipitalis; pne, pneumatic recess; ppo, attachment point of broken processus postorbitalis; qdj, os quadratojugale; rcc, recessus columellae; rps, rostrum parasphenoidale; rtd, recessus tympanicus dorsalis; slp, shelf-like lateral projection formed by crista nuchalis transversa; tbb, tuberculum basilare; vag, foramen nervi vagi. The scale bars equal 10 mm

The caudal portion of the internarial bar (Fig. 2C, G, H is mediolaterally broad and dorsoventrally narrow as in the Sulidae. The caudal nasal bar (Fig. 2A) is slenderer than in crown group Suliformes. Unlike in the partial plotopterid skull SMF Av 599, which was described by Mayr et al. (2015), there is no longitudinal neurovascular sulcus along the lateral surface of the rostrum, just dorsal to the crista tomialis (Fig. 2B). The maxillary bones are co-ossified along their entire length. The processus maxillopalatini are greatly enlarged and likewise co-ossified as they are in all crown group Suliformes but not in sphenisciforms. The ventral surface of the rostrum exhibits a pair of prominent longitudinal ridges (Fig. 3D, 5A), which extend parallel to the cristae tomiales (primary cutting edges of the rostrum) and do not occur in extant Suliformes. Rather than being essentially flat as in crown group Suliformes, the ventral surface of the rostrum forms a shallow midline sulcus between these ridges. Unlike in the Suloidea but as in the Fregatidae, the caudal portions of the maxillaries are laterally slanted. The caudal portion of the massive jugal bar is dorsoventrally deep. As in most neornithine birds, os jugale and os quadratojugale are co-ossified. Here, we note that this is not the case in the Sulidae, in which both bones are separated by a suture (Fig. 6E); to the best of our knowledge, this unusual condition has not been mentioned before.

The neurocranium is proportionally longer than in extant Fregatidae. The size of the orbit corresponds to that of the Sulidae, whereas the orbits of the Anhingidae and Phalacrocoracidae are proportionally shorter relative to the length of the neurocranium. The interorbital section of the neurocranium has a similar width to that of the Sulidae and Phalacrocoracidae. There are no supraorbital fossae for salt glands and these are likewise not discernible within the orbits. A distinct, dorsoventrally extending intermuscular line in the caudal portion of the orbit (Fig. 6A, D) presumably formed the rostral delimitation of musculus pseudotemporalis superficialis, as it does in the Sulidae. Another muscular attachment site is indicated by a fossa rostral to the foramen nervi maxillomandibularis (Fig. 6A, D). The processus postorbitales are broken on both sides of the skull of SMF Av 671, but they are long and laterally projected—similar to the condition in the Fregatidae, Sulidae, and Paleocene stem group sphenisciforms (Mayr et al. subm.)—in a plotopterid skull from the late Oligocene of Japan, which was figured by Kawabe et al. (2014: Fig. 1B) and Knoll and Kawabe (2020: Fig. 5).

The rostrum parasphenoidale is a long and mediolaterally narrow bar; in the plotopterid skull figured by Knoll and Kawabe (2020: Fig. 5), it ventrally delimits a very large fonticulus interorbitalis (as in the Suloidea but unlike the Fregatidae). Furthermore, as in the Suloidea and unlike the Fregatidae, the caudal portion of the rostrum parasphenoidale forms a steep ridge. Unlike in early Paleogene stem group Sphenisciformes (Mayr et al. subm.), there are no basipterygoid processes. The fossae temporales are rostrocaudally wide and medially separated by a long and sharply defined crista nuchalis sagittalis (as in the Phalacrocoracidae, whereas the temporal fossae are proportionally smaller in the Sulidae and—even more so—Anhingidae). As noted by Mayr et al. (2015), the rostral margin of the fossae temporales is sigmoidally curved, which is visible on the left side of the specimen. The crista nuchalis transversa forms a shelf-like lateral projection, which is partially preserved on the left side of the specimen. The crista temporalis is an inconspicuous ridge. As in the Fregatidae and Sulidae, the processus paroccipitales are caudoventrally protruding (in the Anhingidae and Phalacrocoracidae they are more caudally projected).

In caudal view, the neurocranium shows a close resemblance to that of Paleocene stem group sphenisciforms (see Mayr et al. subm.). The condylus occipitalis is large and has a round outline. As in the Sulidae, the foramen magnum has a subrectangular shape, being dorsoventrally deeper than it is mediolaterally wide. Also as in the Sulidae, the ventral margin of the supraoccipital bone forms a marked step. Our identification of the cranial nerve foramina (Figs. 5 and 6) is tentative and based on comparisons with extant Suliformes. As in other suliforms and most other Neornithes, there are separate foramina for nervus vagus and n. glossopharyngealis, whereas both nerves pass through the same foramen in the Phaethontidae (see Mayr 2018). The foramen for n. vagus is not as greatly enlarged as it is in the Anhingidae (Mayr 2018: Fig. 3h). On the right side of the skull, it appears to be represented by two tiny foramina, but this is interpreted as an artefact of preservation, with varnish obscuring the actual foramen. The foramina for nervus hypoglossus are not clearly discernible (the aforementioned tiny foramina are in the wrong place to be foramina nervi hypoglossi).

The basicranial area exhibits a distinctive feature that characterizes sphenisciforms and is absent in suliforms, that is, a marked, arcuate rostral ridge, which delimits an essentially flat lamina parasphenoidalis. Caudally, this ridge is continuous with the tubercula basilaria. An arcuate ridge is also present as an inconspicuous faint line in the Phalacrocoracidae, Anhingidae, and Pelecanidae; the condition in the Sulidae is uncertain because of the apomorphic morphology of the corresponding area of the basicranium.

The otic region, which experienced some damage (before being fossilized) on both sides of the skull, is most similar to that of the Sulidae (Mayr 2020). There is a large, subovate cotyla quadratica otica and a smaller, kidney-shaped cotyla quadratica squamosa. Unlike in sphenisciforms but as in the Suliformes, these cotylae are separated by a large recessus tympanicus dorsalis, which is of similar size to the dorsal tympanic recess in the Sulidae and much larger than that of the Anhingidae and Phalacrocoracidae. As in all crown group Suliformes but unlike in crown group Sphenisciformes, tubae auditivae are absent. There is a shallow sulcus for arteria ophthalmica externa; situated in the midsection of this sulcus is a foramen for the ramus occipitalis of arteria ophthalmica externa (Fig. 6B, C). The sulcus for arteria carotis cerebralis is bridged by a thin bony lamella as it is in the Sulidae and Phalacrocoracidae. As in crown group Suliformes, the foramen nervi maxillomandibularis is large; ventral to it, there is a ridge-like vestige of the ala parasphenoidalis on the right side of the specimen. The recessus columellae is somewhat smaller than the foramen nervi maxillomandibularis. In extant Sulifomes, the recessus columellae is poorly developed in the Fregatidae, in which fenestra vestibuli and fenestra cochleae are well exposed in the otic region (Fig. 6G).

The partial furcula associated with the skull has strap-like rami and a dorsoventrally deep extremitas sternalis, whose cranial surface bears a large facet for the articulation with the carina of the sternum (Fig. 1D, E). A deep extremitas sternalis also occurs in the Fregatidae, whereas the sternal extremity is much narrower in crown group Suloidea.

The proximal end of the humerus exhibits a marked, elongate fossa between the fossa pneumotricipitalis and the tuberculum dorsale (Fig. 1F), which has not yet been reported from other plotopterids in which the humerus is known. Otherwise, and apart from a smaller size, the morphology of the bone does not depart from a proximal humerus which was assigned to ?*Klallamornis clarki* by Mayr and Goedert (2022). The bone exhibits a small, convex crista bicipitalis (Fig. 1G). As in other plotopterids (Mayr and Goedert 2022: Fig. 5), there is a distinct tubercle for the insertion of musculus latissimus dorsi, pars cranialis on the caudal surface of the bone, distal to the fossa pneumotricipitalis. The bone exhibits numerous small boreholes resembling those of the bone-eating marine annelid *Osedax*, which have been previously reported in plotopterid bones from the Pysht Formation (Kiel et al. 2011).

Rib fragments preserved in SMF Av 671 exhibit fused processus uncinati.

## Discussion

### Phylogenetic implications

The morphology of the new skull corroborates suliform affinities of the Plotopteridae and a sister group relationship to the Suloidea. Contrary to previous observations based on fragmentary plotopterid skulls from the Pysht Formation (Mayr et al. 2015), the nostrils of SMF Av 671 are reduced to small openings, as they are in all Suliformes, and are situated at the caudal end of a marked longitudinal sulcus (which was erroneously interpreted as a slit-like narial opening by Mayr et al. 2015). In addition, and as in the Fregatidae and Sulidae, there is a large recessus tympanicus dorsalis, which—according to current phylogenies that indicate an early diverging position of the Fregatidae and Sulidae within Suliformes (e.g., Prum et al. 2015; Kuhl et al. 2021; Stiller et al. 2024)—is secondarily reduced in the Anhingidae and Phalacrocoracidae. Skull features, which support an assignment of plotopterids to the Suloidea include caudally projected processus paroccipitales, prominent tubercula basilaria, and a densely pitted ventral surface of the tip of the rostrum (Fig. 5A).

Some plesiomorphic characters corroborate a position of plotopterids outside crown group Suloidea. In addition to the previously reported (Mayr et al. 2015) presence of a lamella choanalis of the palatine bone, the new skull shows that the nostrils of plotopterids are less reduced than in crown group Suloidea and the longitudinal sulci along the rostrum are much more pronounced; furthermore, the crista bicipitalis of the humerus is not as strongly proximodistally elongated as it is in crown group Suloidea (Fig. 1J, K).

SMF Av 671 agrees with the Phalacrocoracidae in the morphology of the temporal region, which features large fossae temporales and a long crista nuchalis sagittalis. However, these similarities are likely to be plesiomorphic for the Suloidea, because plotopterids are outside the clade formed by the Anhingidae and Phalacrocoracidae, with the latter two taxa sharing a reduced recessus tympanicus dorsalis and a characteristic laterally bulging neurocranium in the area of the parietal bones (Fig. 3J, L), as well as broadly rounded (rather than pointed) processus paroccipitales.

**Fig. 7:**
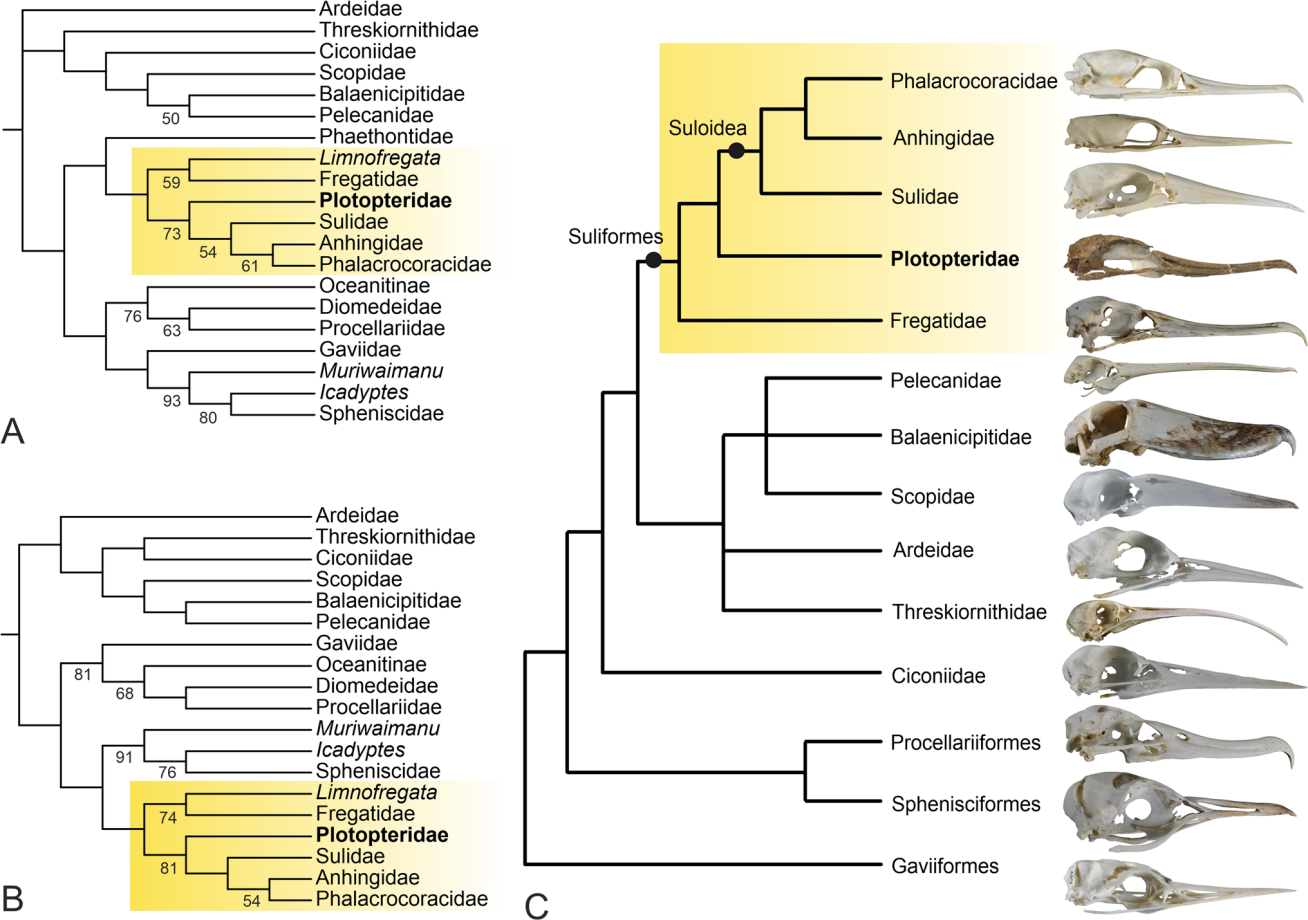
The phylogenetic affinities of plotopterids. **A** Strict consensus tree of four most parsimonious tree resulting from the analysis of the morphological character matrix (length = 260, consistency index = 0.41, retention index = 0.63); bootstrap support values are shown next to the internodes. **B** Single most parsimonious tree resulting from the analysis after exclusion of the Phaethontiformes (length = 245, consistency index = 0.44, retention index = 0.67); bootstrap support values are shown next to the internodes. **C** Consensus phylogeny of the Aequornithes based on nuclear gene sequences (Prum et al. 2015; Kuhl et al. 2021; Stiller et al. 2024), with the Plotopteridae added based on the results of the analysis of the morphological data. Suliformes are highlighted in yellow. In **C**, skulls of representative species are shown

Our analysis of the emended character matrix of Mayr et al. (2015) yielded the same tree topology as the initial study. Plotopteridae resulted as the sister taxon of the Suloidea, and Sphenisciformes were found in a clade together with the Gaviiformes and Procellariiformes (Fig. 7A). Concerning the latter clade and the interrelationships of other extant taxa, the resultant tree does not conform to phylogenies based on nuclear sequence and retroposon data, which find the Gaviiformes to be the sister taxon of all other Aequornithes (the “waterbird clade”; e.g., Kuramoto et al. 2015; Prum et al. 2015; Kuhl et al. 2021; Stiller et al. 2024). This is also true for the position of the Phaethontiformes, which were obtained as the sister taxon of the Suliformes in our analysis, but are placed outside Aequornithes in current sequence-based studies. Other large-scale analyses of morphological data (Livezey and Zusi 2007; Smith 2010) were likewise unable to recover extant taxa of the Aequornithes in positions consistent with strongly supported tree topologies based on nuclear sequence data (Fig. 7C).

After exclusion of the Phaethontiformes, our analysis resulted in a sister group relationship between the Sphenisciformes and Suliformes (Fig. 7B). This contrasts with almost all analyses of nuclear sequence data, which robustly support Sphenisciformes and Procellariiformes as sister groups. However, one analysis of an individual nuclear gene (Fain and Houde 2004) and a large-scale analysis of mitochondrial sequence data (Brown et al. 2008) also found a (Sphenisciformes + Suliformes) clade. Another analysis of mitochondrial genes (Gordon et al. 2021) supported a sister group relationship between the Sphenisciformes and Fregatidae, but did not recover a monophyletic Suliformes. The signal from the above analyses for closer affinities of sphenisciforms to suliforms than to procellariiforms may be readily attributed to inadequate data if it were not for the fact that Paleocene stem group Sphenisciformes differ markedly from procellariiforms in their postcranial osteology and are instead very similar to the Plotopteridae (Mayr et al. 2021, subm.). In particular, there are notable resemblances in the morphology of the hindlimb elements including a short and stocky tarsometatarsus, which are difficult to explain by convergence in wing-propelled divers. SMF Av 671 shows a previously unknown derived skull feature shared by plotopterids and sphenisciforms, that is, a conspicuous arcuate ridge which rostrally delimits the lamina parasphenoidalis (Figs. 5B, C, 6C; see Mayr et al. subm. for the occurrence of this character in Paleocene stem group sphenisciforms).

The Fregatidae and Plotopteridae are successive sister taxa of the Suloidea, so that features shared by both taxa—such as a short and stocky tarsometatarsus—are likely to be plesiomorphic for the Suliformes. A sister group relationship between sphenisciforms and suliforms would, therefore, show some of the similarities of plotopterids and sphenisciforms to be symplesiomorphies. However, if sphenisciforms and procellariiforms are sister taxa—which is strongly supported by current nuclear sequence and retroposon data—the significance of these resemblances remains elusive.

### Evolutionary significance

Improved knowledge of the skull morphology of plotopterids contributes to a better understanding of character evolution in the Suliformes. In its shape and proportions, and apart from a less deeply hooked tip, the rostrum of SMF Av 671 resembles that of the Fregatidae. Because the Fregatidae and Plotopteridae are successive sister taxa of the Suloidea, a fregatid- or plotopterid-like rostrum is likely plesiomorphic for the Suliformes. The rostrum of plotopterids also corresponds to that of some long-beaked phalacrocoracid species (*Phalacrocorax* spp., *Leucocarbo* spp.), whereas the derived morphologies of the straight and pointed rostrum of the Anhingidae and Sulidae are due to the specialized foraging methods of these birds, that is, spearing prey items (Anhingidae) and plunge diving (Sulidae).

The earliest stem group Sphenisciformes had long and straight beaks (Fig. 4G), and the differences in beak morphology indicate that the foraging ecology of plotopterids differed from that of early Cenozoic penguins, which are considered to have speared prey items (Chávez-Hoffmeister 2020). By contrast, the hooked tip of the rostrum of plotopterids suggests that these birds were snatching prey items in underwater pursuit. The longitudinal ridges along the ventral surface of the rostrum, which are unknown in other birds and constitute an autapomorphy of plotopterids, probably enhanced the grasping capabilities of the beak.

The different bill morphologies of plotopterids and early Paleocene penguins may have been due to disparate marine habitats along with differing prey and methods of prey pursuit. It was hypothesized that the evolution of plotopterids was associated with the origin of kelp in the North Pacific (Mayr and Goedert 2022; Kiel et al. 2024). The “underwater forests” formed by kelp house diverse ecosystems and provide shelter for numerous marine organisms (Teagle et al. 2017), which can be exploited by foraging seabirds that use their beaks to grasp prey. By contrast, early Paleogene penguins are likely to have chased fishes in open water, which may have favored the evolution of beak morphologies that reduced hydrodynamic drag and enabled these birds to swim fast and spear larger prey items.

A notable feature of plotopterids is the presence of marked sulci along the lateral surfaces of the rostrum, which are less pronounced in all extant Suliformes. However, the stem group frigatebird *Limnofregata* likewise has marked nasal sulci, which were interpreted as slit-like nostrils (Olson 1977; Olson and Matsuoka 2005). In light of the rostrum morphology of plotopterids, this latter hypothesis needs to be re-evaluated and it appears possible that the nostrils of *Limnofregata* were likewise restricted to the caudal portions of these sulci (their exact extent is not clearly discernible in the published specimens of *Limnofregata*). Because greatly elongated nostrils occur in juvenile Suliformes (Olson 1977; Mayr 2005; Stucchi 2013), it is possible that they were present in the suliform stem species, and the nasal sulci of plotopterids are best interpreted as vestiges of such long nostrils. If they were present in *Limnofregata*, long nostrils were reduced two times independently within Suliformes, in the Fregatidae and in the Suloidea. Very long and slit-like nostrils are also found in the earliest stem group Sphenisciformes (Mayr et al. 2017, subm.), so that this feature may be a plesiomorphic trait of a more inclusive clade of the Aequornithes.

Unlike suliform birds, sphenisciforms have enlarged supraorbital nasal glands which assist in the excretion of excessive salt. Future studies will have to assess whether the nostrils are reduced in suliforms to prevent salt water influx into the nasal cavity and whether there is a functional correlation between the size reduction of the nostrils and the lack of enlarged salt glands.

The caudal portion of the rostrum of SMF Av 671 differs from two partial plotopterid skulls from the Pysht Formation in the dorsoventrally deeper nasal sulcus (Fig. 2A, B). The Pysht Formation fossils are from latest early Oligocene/early late Oligocene strata (about 27‒29 million years ago), and with an age of about 35 million years, the new skull from the late Eocene Lincoln Creek Formation is about 6‒8 million years older. Because these skulls and other avian fossils from the Olympic Peninsula are not affected by diagenetic compression, we consider their morphologies to reflect the actual shape of the nostril and nasal sulcus. The differences between the fossils from the Lincoln Creek and Pysht formations may be due to morphological changes in the evolution of plotopterids, with the fossil from the Lincoln Creek Formation showing a more plesiomorphic condition of the nostril and nasal sulcus. A plotopterid skull from the late Oligocene of Japan (Kawabe et al. 2014; Fig. 1B; Knoll and Kawabe 2020; Fig. 5) corresponds to the specimens from the Pysht Formation, possibly providing further evidence that there was a change in rostrum morphology in the evolution of plotopterids, with pre-Oligocene species having had deeper nasal sulci.

## Data Availability

No datasets were generated or analysed during the current study.
